# Supplementary material for: A causal role for right temporo-parietal junction in signaling moral conflict
Source: eLife. 2018 Dec 18;7:e40671. doi: 10.7554/eLife.40671 (PMC6298767; doi:10.7554/eLife.40671)
Supplement: Supplementary file 1 [file elife-40671-supp1.docx]

***Supplementary File 1.*** *Questionnaire scores for both experimental groups.*

| **Group** | **IPIP** | **Mach IV** | **PSB** | **SIAS** | **SVO** | **BIS** | **MMSQ** |
| --- | --- | --- | --- | --- | --- | --- | --- |
| **TPJ** | 11.80 (.6) | 46.73 (7.9) | 17.90 (1.2) | 44.20 (6.2) | 2.81  (.4) | 61.93 (7.1) | 10.81  (1.7) |
| **Vertex** | 12.00 (.7) | 48.69 (8.4) | 18.57 (2.0) | 46.44 (5.2) | 2.69  (.4) | 61.44 (5.5) | 11.13  (1.3) |
| **p value** | **0.403** | **0.513** | **0.284** | **0.287** | **0.431** | **0.829** | **0.567** |

*IPIP: International Personality Item Pool; Mach IV: Machiavelic test; Prosocial Personality Battery (PSB); Social Interaction Anxiety Scale: SIAS; SVO: Social Value Orientation; BIS: Barratt Impulsivity Scale; Multidimensional Mood State Questionnaire: MMSQ, results shown after cTBS.*
