# Supplementary material for: A causal role for right temporo-parietal junction in signaling moral conflict
Source: eLife. 2018 Dec 18;7:e40671. doi: 10.7554/eLife.40671 (PMC6298767; doi:10.7554/eLife.40671)
Supplement: Supplementary file 2 — A, good organization; B, bad organization. [file elife-40671-supp2.docx]

**Supplementary File 2.** Full regression audience model. A, good organization; B, bad organization.

**A**

|  | **Coefficient** | **Std. Error** | **z** | **P>\|z\|** | **95% Conf. Interval** | |
| --- | --- | --- | --- | --- | --- | --- |
| Subject loss | 0.8096 | 0.0229 | 35.25 | 0.0001 | 0.7646 | 0.8546 |
| Organization gain | 0.1342 | 0.0046 | 28.61 | 0.0001 | 0.1250 | 0.1434 |
| Audience | 0.5177 | 0.2559 | 2.02 | 0.043 | 0.0161 | 1.0192 |
| cTBS | 1.66 | 0.6347 | 2.62 | 0.009 | 0.4193 | 2.9073 |
| cTBSxAudience | 0.0460 | 0.1656 | 0.28 | 0.781 | -0.278 | 0.3706 |
| constant | -0.7988 | 0.4682 | -1.71 | 0.088 | -1.716 | 0.1189 |

Number of obs = 5800; Number of subjects = 29; Obs per group: min = 200; max =200; avg. = 200

Integration points = 7 Wald chi2(4) = 1338.33

Log Likelihood = -1923.72 Prob > chi2 = 0.00001

Estimate: 1.69; Standard error: 0.26; cTBS: group

**B**

|  | **Coefficient** | **Std. Error** | **z** | **P>\|z\|** | **95% Conf. Interval** | |
| --- | --- | --- | --- | --- | --- | --- |
| Subject gain | 0.7395 | 0.0219 | 33.7 | 0.0001 | 0.6965 | 0.7825 |
| Organization gain | -0.1497 | 0.0049 | -30.25 | 0.0001 | -0.1594 | -0.1400 |
| Audience | -0.1304 | 0.2599 | -0.5 | 0.616 | -0.6398 | 0.3789 |
| cTBS | -0.8991 | 0.7320 | -1.23 | 0.219 | -2.3338 | 0.5355 |
| cTBSxAudience | -0.0883 | 0.1659 | -0.53 | 0.594 | -0.4137 | 0.2369 |
| constant | -0.7473 | 0.5364 | -1.39 | 0.164 | -1.7987 | 0.3040 |

Number of obs = 5800; Number of subjects = 29; Obs per group: min = 200; max =200; avg. = 200

Integration points = 7 Wald chi2(4) = 1294.48

Log Likelihood = -1909.99 Prob > chi2 = 0.00001

Estimate: 1.64; Standard error: 0.21; cTBS: group
