# Supplementary material for: A causal role for right temporo-parietal junction in signaling moral conflict
Source: eLife. 2018 Dec 18;7:e40671. doi: 10.7554/eLife.40671 (PMC6298767; doi:10.7554/eLife.40671)
Supplement: Supplementary file 3 — A, good organization; B, bad organization. [file elife-40671-supp3.docx]

**Supplementary file 3.** Full regression model. A, good organization; B, bad organization.

**A**

|  | **Coefficient** | **Std. Error** | **z** | **P>\|z\|** | **95% Conf. Interval** | |
| --- | --- | --- | --- | --- | --- | --- |
| Subject loss | 0.1248 | 0.0137 | 9.1 | **0.0001** | 0.0979 | 0.1517 |
| Organization gain | 0.6046 | 0.0660 | 9.16 | **0.0001** | 0.4752 | 0.7339 |
| Audience | 0.3487 | 0.2491 | 1.4 | 0.162 | -0.1396 | 0.8370 |
| cTBS | 0.1790 | 0.4312 | 0.42 | 0.678 | -0.6661 | 1.0242 |
| cTBSxAudience | 0.1927 | 0.1630 | 1.18 | 0.237 | -0.1267 | 0.5121 |
| cTBSxSubject loss | 0.0037 | 0.0090 | 0.41 | 0.681 | -0.0139 | 0.0213 |
| cTBSx Organization gain | 0.1358 | 0.0446 | 3.04 | **0.002** | 0.0483 | 0.2233 |
| constant | -0.5810 | 0.7642 | -0.76 | 0.447 | -2.0787 | 0.9167 |

Number of obs = 5800; Number of subjects = 29; Obs per group: min = 200; max =200; avg. = 200

Integration points = 7 Wald chi2(4) = 1327.33

Log Likelihood = -1915.32 Prob > chi2 = 0.00001

Estimate: 1.48; Standard error: 0.19; cTBS: group

**B**

|  | **Coefficient** | **Std. Error** | **z** | **P>\|z\|** | **95% Conf. Interval** | |
| --- | --- | --- | --- | --- | --- | --- |
| Subject loss | -0.1864 | 0.0154 | -12.14 | **0.0001** | -0.2165 | -0.1563 |
| Organization gain | 0.5836 | 0.0659 | 8.86 | **0.0001** | 0.4545 | 0.7128 |
| Audience | -0.1512 | 0.2559 | -0.59 | 0.555 | -0.6527 | 0.3503 |
| cTBS | -0.4857 | 0.4243 | -1.14 | 0.252 | -1.3174 | 0.3459 |
| cTBSxAudience | -0.0837 | 0.1635 | -0.51 | 0.609 | -0.4041 | 0.2367 |
| cTBSxSubject gain | 0.0263 | 0.0096 | 2.74 | **0.006** | 0.0075 | 0.0450 |
| cTBSx Organization gain | 0.1089 | 0.0432 | 2.52 | **0.012** | 0.0243 | 0.1935 |
| constant | -0.3884 | 0.7912 | -0.49 | 0.624 | -1.9392 | 1.1624 |

Number of obs = 5800; Number of subjects = 29; Obs per group: min = 200; max =200; avg. = 200

Integration points = 7 Wald chi2(4) = 1294.48

Log Likelihood = -1909.99 Prob > chi2 = 0.00001

Estimate: 1.58; Standard error: 0.20; cTBS: group
